# Supplementary material for: Complexome profiling of the Chlamydomonas psb28 mutant reveals TEF5 as an early PSII assembly factor
Source: Plant Cell. 2025 Mar 17;37(6):koaf055. doi: 10.1093/plcell/koaf055 (PMC12164590; doi:10.1093/plcell/koaf055)
Supplement: koaf055_Supplementary_Data [file koaf055_supplementary_data.zip › psb28_interactive.zip!/plots/intensity/qualityPlots/peptides/rpl14_Cre-1.g2717058.t1.p1.html]

### Cre-1.g2717058.t1.p1

Trivial name: rpl14  
SeqCoverage [median]: 23.00  
Mass: 13.45 kDa  
  
 

| Sequence | MissedCleavage | Mass [Da] | wtIntensity | muIntensity | Score |
| --- | --- | --- | --- | --- | --- |
| FDDNAAVIINK | 0 | 1218.62 | 0.00 | 534.42 | 87.81 |
| MIKPLSYLNVADNSGAR | 1 | 1847.96 | 0.00 | 1547.16 | 56.41 |
|  |  |  | 0.0 | 2081.6 |  |
